# A Series of TA-Based and Zero-Background Vectors for Plant Functional Genomics

**DOI:** 10.1371/journal.pone.0059576

**Published:** 2013-03-29

**Authors:** Chuntao Wang, Xianlun Yin, Xiangxiang Kong, Wansha Li, Lan Ma, Xudong Sun, Yanlong Guan, Christopher D. Todd, Yongping Yang, Xiangyang Hu

**Affiliations:** 1 Key Laboratory of Biodiversity and Biogeography, Kunming Institute of Botany, Chinese Academy of Science, Kunming, Yunna, China; 2 Plant Germplasm and Genomics Center, the Germplasm Bank of Wild Species, Kunming Institute of Botany, Chinese Academy of Sciences, Kunming, Yunna, China; 3 University of the Chinese Academy of Sciences, Beijing, China; 4 Department of Biology, University of Saskatchewan, Saskatoon, Canada; Nanjing Agricultural University, China

## Abstract

With the sequencing of genomes from many organisms now complete and the development of high-throughput sequencing, life science research has entered the functional post-genome era. Therefore, deciphering the function of genes and how they interact is in greater demand. To study an unknown gene, the basic methods are either overexpression or gene knockout by creating transgenic plants, and gene construction is usually the first step. Although traditional cloning techniques using restriction enzymes or a site-specific recombination system (Gateway or Clontech cloning technology) are highly useful for efficiently transferring DNA fragments into destination plasmids, the process is time consuming and expensive. To facilitate the procedure of gene construction, we designed a TA-based cloning system in which only one step was needed to subclone a DNA fragment into vectors. Such a cloning system was developed from the pGreen binary vector, which has a minimal size and facilitates construction manipulation, combined with the negative selection marker gene *ccdB*, which has the advantages of eliminating the self-ligation background and directly enabling high-efficiency TA cloning technology. We previously developed a set of transient and stable transformation vectors for constitutive gene expression, gene silencing, protein tagging, subcellular localization analysis and promoter activity detection. Our results show that such a system is highly efficient and serves as a high-throughput platform for transient or stable transformation in plants for functional genome research.

## Introduction

With the development of sequencing technology, particularly the secondary generation of sequencing technology, which includes Solexa and 454, it is not very difficult to decode complex eukaryotic genomes. However, with the increase of available organ genome information, uncovering the mysteries behind the genome and deciphering gene function is a huge challenge. For example, in the model plant *Arabidopsis*, it has been estimated that there are approximately 30,000 genes necessary for viability, and rice has more genes than *Arabidopsis* [1]. Generally, generating a transgenic plant by ectopic expression or silencing of the target gene of interest is a typical method to investigate target gene function. Other approaches, including promoter analysis, protein subcellular localization, site-directed mutagenesis, and in vitro and in vivo biochemical analysis, also provide more information for understanding gene function [2]. Nevertheless, all of the approaches mentioned above require molecular manipulations, such as protein fusion and gene modification, and strongly depend on restriction enzyme digestion and ligation. Such procedures always lead to increased time consumption and experimental expense. In particular, we must consider how to select the appropriate enzyme during construction, but not all restriction enzymes are readily available. Thus, gene manipulation and modification have already become the bottleneck problem for large-scale functional genomic research in plants. Developing an efficient and cost-efficient approach for gene manipulation has become compulsory for us.

Using restriction enzymes for gene recombination is direct and efficient; such technique is also limited by the potential presence of the restriction sites within the target gene. Recently, other approaches, such as the Gateway cloning system from Invitrogen and the Creator cloning system from Clontech, have resulted in revolutionary effects on gene recombination and manipulation [3], [4]. These cloning systems can be divided into two steps. The first step is to insert the target gene into the donor vector at a particular location flanked by two specific recombination sites. The second step is to recombine the target gene in the donor vector into the various destination vectors by use of a site-specific recombination reaction and LR clonase. Unlike a typical restriction enzyme-dependent recombination system, the Gateway cloning system is independent of restriction enzymes and ligases and does not require consideration of potential restriction enzyme digestion sites inside the target gene. The BP and LR reactions are also highly efficient. The downstream destination vector has already become a public resource and is easy to access. High-throughput gene construction based on this technology has become possible; thus, such technology is widely used by researchers. However, the limitations of this technology cannot be ignored. For example, a two-step manipulation continues to be time and labor consuming. The patented BP and LR enzymes are very expensive, and the long primers containing the specific attachment site (att) occasionally add to the difficulty of gene amplification. In addition to the Gateway system, other high-throughput cloning systems, such as the LIC system [5], have been developed. However, similar problems, including primers that are too long, still exist.

A TA-based cloning system is the simplest and most efficient method for cloning PCR-amplification products. The 3′-A-end PCR products can be ligated into a 3′-T-end linear plasmid using T4 DNA ligase [6], [7], [8]. It is not necessary to consider potential restriction enzyme digestion sites existing in the DNA fragments, and recombinase or topoisomerase for site-specific recombination are not required. Thus, attempts have been made to apply TA-based cloning strategies to gene construction other than PCR products, but typical TA-based cloning systems can only be used with a specific TA cloning vector. The white-blue screen system for positive clone screening is not very stable or stringent, and it is affected by the culture environment and insertion fragments. The ccdB gene encoding the bacterial suicide protein is already widely used as the negative selection marker in molecular manipulations because it has zero background during screening [8]. Here, we report a TA-based expression system in which the 3′-T-end of the vector was generated by *Xcm*I digestion. To eliminate possible self-ligation of vectors, we introduced the ccdB gene, a lethal gene for *E.coli* DH 5α [8], into the TA-base expression system. To improve the efficiency of the cloning procedure, we selected pGreen 0029, which has a minimal size and high copy numbers, as the backbone [9]. Our results demonstrated the efficiency of this cloning procedure in developing a set of stable transformation vectors for constitutive, inducible gene expression, gene silencing, and protein subcellular localization and promoter activity analysis. Thus, our system is a convenient and versatile cloning system and could represent another choice for researchers in gene construction.

## Materials and Methods

### TA-based pGreen Vector Construction and Propagation

The pGreen vector, pZaBaTA [10], pLB12 [3] and pLIC [5] series vectors are available from the *Arabidopsis* Stock Centre (www.arabidopsis.org). Standard molecular manipulations were performed to make the constructs. All plasmids derived from PCR products were verified by sequencing. All primers used for making constructs, insertions, sequencing or RT-PCR are shown in Table S1. The fragments of 35S/Ubi-CCD-NOS, 35S/Ubi-HA-CCD-NOS, 335S/Ubi-Myc-CCD-NOS, 35S/Ubi-Flag-CCD-NOS, 35S/Ubi-GFP-CCD-NOS and 35S/Ubi-DsRed-CCD-NOS were amplified from the pZabaTA vector system using the indicated primers (Table S1) and then subcloned into pGreen by *KpnI/SacI* digestion to form the vectors pGreen-35/Ubi-B/K or pGreen-35/Ubi-HA/Flag/Myc/GFP/DsRed-B/K. For the pGreen- 3GFP/Gus/sYFP/DsRed/tdTomato-B/K vectors, the fragment of XcmI-CCD-XcmI was amplified from the pZabaTA vector using the indicated primers and then cloned into the pGreen vector by SacI/SpeI digestion. 3GFP/GUS/sYFP/DsRed/tdTomato-NOS fragments were amplified from pLIC vectors using the indicated primers and then cloned into the KnpI/SpeI sites of pGreen. For inducible vectors, the OlexA TATA fragment was amplified from the pLB12 vector using the indicated primers and inserted into the SacI/SpeI sites of the pGreen vector. Then, the HA-CCD-NOS fragment was amplified from the pZaBaTA vector and inserted into the SpeI/XhoI sites of pGreen. Finally, the Mini35S-XVE-Nos fragment was amplified from the pLB12 vector and inserted into the XhoI/KpnI site of pGreen to form the vector pGreen-XVE-B/K.

### TA Cloning Using Modified pGreen Vectors

TA-based modified pGreen vectors were digested with XcmI (New England Biolabs). PCR products were amplified using the proofreading pfu DNA polymerase (Takara, Japan), followed by the A-addition procedure previously described. The digested pGreen vector and PCR-amplified product were purified from agarose gels with the Qiagen MinElute Gel Extraction Kit. The ligation was carried out using T4 DNA ligase from NEB (New England Biolabs) in a total volume of 10 µl containing 50 ng of T-vector and the corresponding volume of PCR product with a standard insert-to-vector molar ratio of 3∶1. The ligation reaction mixture was transformed into *E. coli* strain DH5a by electroporation. All recombinant plasmids identified from individual *E. coli* colonies were verified by sequencing. The PCR products of SOS2, ICE1 and CBL5 were amplified with the primers listed in Table S1.

### Plant Growth Conditions, Gene Transformation and Basta Selection

*Arabidopsis thaliana* (Columbia background) was grown in pots in a Greenhouse at high humidity over a 16-hr photoperiod under fluorescent lamps at a light intensity of 150 to 300 µE m^−2^ sec^−1^. *Agrobacterium*-mediated transformation of *Arabidopsis thaliana* was performed by the floral dip method [11], and transgenic lines were selected by spraying 50 mg/L Basta solution on the two-cotyledon seedling growing in pots every week for a total of three times, or screened on a 10 cm Petri-dish with an additional 35 mg/L kanamycin. Transgenic lines were further confirmed by PCR using the corresponding primers.

### Transient Expression in *Arabidopsis* and Rice Mesophyll Protoplasts and Fluorescence Microscopy Assays

The transient expression method was performed according to a previously reported method [12]. Approximately 10 µg of plastid was used to transfect the protoplast of arabidopsis or rice, mediated by PEG4000. The transfection was stopped after incubating for 20 min. The transfected protoplast was incubated in the dark for 16 hours. Fluorescence of the GFP chimeric gene was detected by an Olympus FluoView® confocal microscope with excitation and emission filters of 450–490 nm and 520–560 nm, respectively.

### GUS Staining

Ten-day-growth T1 seedlings were collected for a GUS assay. The GUS staining assay was performed according to previously reported methods [12], [13]. Briefly, fresh seedlings were put into a staining solution containing 0.5 mg/ml X-Gluc in PBS (pH 7.0), 10 mM Na2EDTA, 0.5 mM K^+^ ferricyanide, 0.5 mM K^+^ ferrocyanide and 0.06% Triton X-100 overnight at 37°C, and depigmentation of the sample was performed by transferring into a gradient concentration of alcohol (30%, 75%, and 95%).

### Gene Expression Analysis by Quantitative Real-time RT-PCR and Small RNA Northern Blotting

Total RNA was extracted as described previously [14]. After the total RNA was extracted, DNA-free total RNA was used for first-strand cDNA synthesis in a 20-µL reaction volume containing 2.5 units of avian myeloblastosis virus reverse transcriptase XL (Takara, Japan) and 1 µL of oligo(dT) primers. For real-time PCR, cDNA aliquots of 1 µL were used for each quantitative PCR reaction in total 20 µL reaction system. For quantification with the ABI PRISM 7300 sequence diction system (Applied Biosystems), the Absolute QPCR SYBR GREEN mix (Thermo Fisher) was used according to the manufacturer’s instructions. Gene-specific primers (100 nM, listed in Table S1) were used for qRT-PCR. The thermal treatment was 15 min at 95°C, followed by 40 cycles of 15 s at 30 s at 62°C and 30 s at 72°C. Single product amplification was validated by melting curve analysis.

For small RNA detection, total RNA was extracted with Trizol reagent (Invitrogen). Approximately 50 µg of total RNA was separated on a 15% PAGE gel and then transferred to a nylon membrane. miR319 and miR171 were detected by probes complementary to the miR319 and miR171 loop sequences, which were modified with 3′ and 5′ biotin. After stringent washes, signals on the membrane were detected according to the manufacturer’s instructions for the chemiluminescent nucleic acid detection module (Thermo).

### Protein Extraction and Western Blotting

Plant seedlings (2 g) were homogenized in 10 ml of ice-cold isolation medium (250 mM mannitol, 25 mM Hepes-Tris, p H7.4, 1 mM EDTA, 1% (w/v), polyvinylpyrrolidone, 10% v/v glycerol, and 1 mM dithiothreitol) at 4°C. The homogenate was filtered through four layers of cheesecloth and centrifuged at 1500 g for 30 min at 4°C. The supernatant was used for western blotting. A 50 µg sample of supernatant protein was separated by SDS-PAGE using a 12% acrylamide resolving gel (Mini Protein III system, Bio-Rad) as described previously [14]. The separated proteins were then transferred to polyvinylidene difluoride membrane, probed with the anti-HA antibody (Roche, Germany) at 1∶3000, and then probed with a horseradish peroxide-conjugated goat anti-mouse secondary antibody (Promega). The resultant signal was detected using an ECL kit (GE healthcare, USA).

## Results

### Construction of TA-based Expression System

**Figure 1:**
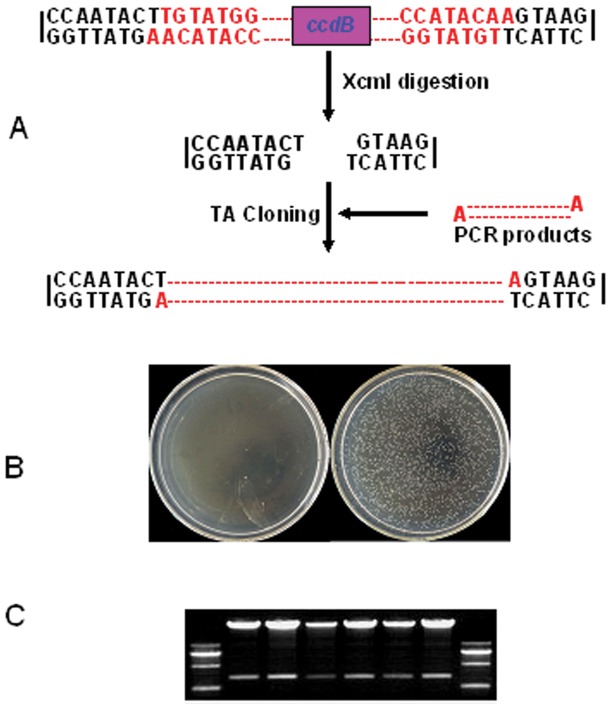
Construction of the TA-based expression vectors. A, workflow for the direct cloning of PCR products using the TA-based expression vector system. TA-based expression vectors were digested by XcmI to produce 3′-T-overhangs. The PCR products were ligated with the XcmI-digested vector by T4 ligase. B, Left: Self-ligation of the XcmI-digested vector by T4 ligase. Right: Ligation of the PCR product of the GFP gene with the XcmI-digested vector by T4 ligase, followed by transformation into DH5α strains. C, Samples of restriction digestion analyses of randomly selected colonies from the ligation of the XcmI-digested pGreen vector with the product of the GFP gene using T4 ligase.

TA cloning has been widely used for gene construction because of its simplicity and high efficiency. There are two fundamental methods to produce T-vectors. One method is to add “T” at the blunt end of a linear plasmid by *Taq* DNA polymerase or terminal deoxynucleotidyl transferase. This method has a defect in that the efficiency of adding T-ends is very low, as many self-ligation products are produced. Another method to produce T-overhangs is mediated by restriction enzymes, such as *Ahd*I, *BciV*I, *Bfi*I, *Hph*I, *Mnl*I, *Taa*I, and *Xcm*I. Of these enzymes, *Xcm*I has been widely used because of its superior digestion efficiency [15], [16]. Although using restriction enzymes to generate T-overhangs is efficient, self-ligation cannot be ruled out because of the potential for incomplete enzyme digestion. To eliminate potential self-ligation, the *ccd*B gene, which is widely applied in the Gateway cloning system from Invitrogen, was used (Figure 1A). The protein produced by this gene causes lethality to *E. coli* strain DH5α by interfering with the activity of DNA gyrase, but it is not lethal to *E. coli* strain DB3.1. To check the efficiency of our TA-based pGreen expression vectors, ligation products of *Xcm*I-digested pGreen-CCD (Figure 1B) with or without PCR products of GFP were transformed to *E. coli* strain DH5α. The results showed that only the ligation products of *Xcm*I-digested pGreen-35S-CCD with a GFP fragment could survive in *E. coli* strain DH5α (Figure 1C). To further assess the ligation efficiency, we selected several colonies randomly and extracted the plasmid to perform restriction enzyme tests. Figure 1C shows that all of the colonies contained the GFP fragment, indicating that the success rate of ligation using our system was 100%. These results suggested that the *ccd*B gene worked effectively in our TA-based pGreen system.

### TA-based Expression Vectors Using the pGreen Backbone

**Figure 2:**
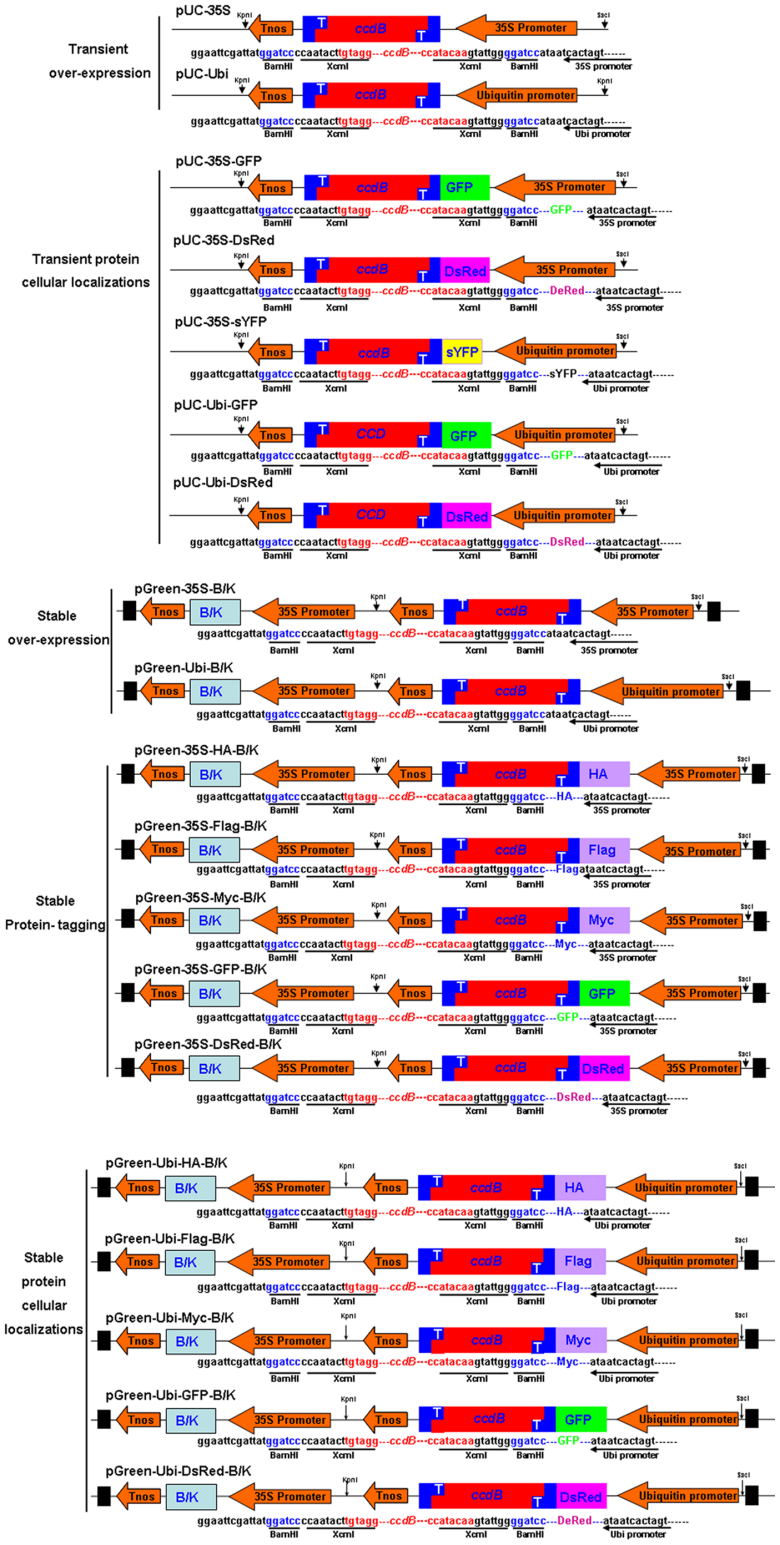
TA-based expression vectors for gene overexpression, protein subcellular localization, protein tagging, promoter analysis and inducible expression in *Arabidopsis*. A, Schematic structures of TA-based transient expression vectors generated by XcmI digestion. B, Schematic structures of Agrobacterium-mediated stable transformation vectors generated by XcmI digestion. The dark boxes at the left and right ends signify the T-DNA LB and RB borders.

Plasmid size and copy number in *E. coli* are the two main limiting factors that affect the efficiency of *in vitro* recombination manipulation. In our report, We developed a set of transient and stable expression vectors for different application in both dicot and monocot plants. The backbone of all the transient expression vectors is derived from pUC vector, a small-size (about 3 kb) and high-copy-number cloning vector that can facilitate the isolation of a large amount of plasmid DNA for transient expression. For stable expression, we selected pGreen0029 (Basta resistance) and pGreen029 (Kanamycin resistance) vectors, both belonging to the pGreen series of vectors, as backbones of TA-based expression vectors. The pGreen vector series has only approximately 4.6 kb of length, with a high copy number in *E. coli* strains. These characteristics were convenient for the gene construction procedure. Using the pUC, pGreen0029 and pGreen029 vectors, we constructed a set of transient, stable and inducible expression vectors for dicot (35 promoter) and monocot (ubiquitin promoter) plant transformation. The backbone of all of the transient and stable vectors was derived from the pGreen vector (website: www.pGreen.com), which eliminates background differences between vectors during transient and stable transformation. The high copy number of these vectors provides the advantage of obtaining a large amount of plasmid DNA for transient and stable expression and further cloning construction. For transgenic plants, the 35S promoter is more efficient in dicots, such as tobacco and Arabidopsis, while the ubiquitin promoter is more effective in monocots, such as rice and maize. In our vector system, the 35S and ubiquitin promoters were used for dicots (arabidopsis) and monocots (rice), respectively. For phenotypic analysis, we constructed the pGreen-35S/ubiquitin-CCD vector to transiently or stably overexpress the interested genes and fused commercial tags (HA, Myc and FLAG) to facilitate western blotting assays with their respective antibodies (Figure 2). For protein subcellular location analysis, we fused the protein of interest with GFP or DsRed to observe the subcellular location of the fluorescent protein (Figure 2). For promoter analysis, we used GFP, 3xGFP, DsRed, GUS, sCFP or tdTomato as the reporter (Figure 2). Inducible vectors, including tissue-specific and constitutively induced vectors, were also constructed. These vectors should meet with the need of most researchers who conduct gene functional research in plants.

### Measuring Target Protein Subcellular Localization by Transient and Stable Vector Systems

**Figure 3:**
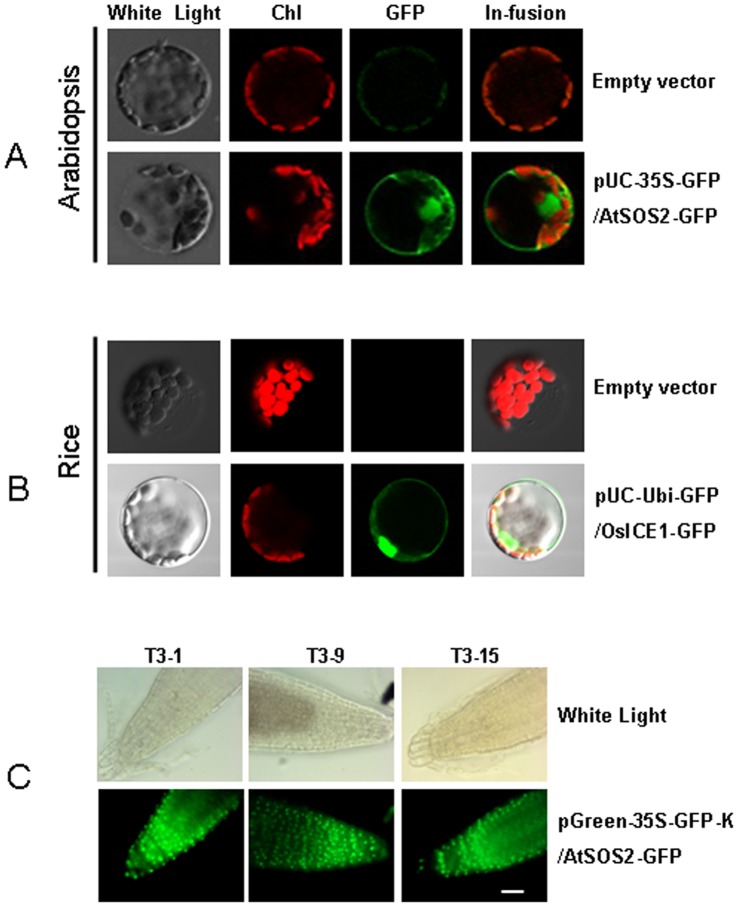
Subcellular protein localization using TA-based expression vectors. A&B, Transient expression of 35S promoter lined with GFP-fused AtSOS2 protein in *Arabidopsis* mesophyll protoplasts(A), or Ubiquitin promoter linked GFP-fused OsICE1 protein in rice mesophyll protoplasts (B) visualized by fluorescence microscopy. Top: mesophyll protoplasts transformed with empty vectors, Bottom: mesophyll protoplasts transformed with the vector with AtSOS2-GFP (A) or OsICE1-GFP (B). C, Overexpression of the AtSOS2-GFP fusion protein in the root tips of transgenic Arabidopsis by the TA-based pGreen -35S-K expression vector. T3-1, T3-9 and T3-15 indicate three individual T2 transgenic lines overexpressing the AtSOS2-GFP fusion protein.

We divided our vector system for overexpressing genes of interest into two parts. The first part contains a set of vectors for transient transformation. The second part is for stable transgenic plants. Both contain the 35S or ubiquitin promoter for dicot or monocot plant transformation, respectively. To evaluate the efficiency of TA-based expression vectors for cellular localization, we constructed transient vectors by fusion of the AtSOS2 protein (a Ser/Thr protein kinase involved in plant responses to saline stress) to GFP drived by 35S promoter in pUC-35S-GFP vector. Meanwhile, we also make the construct containing OsICE1 protein (a bHLH transcriptional protein involving in cold stress) to GFP drived by ubiquitin promoter in pGreen-Ubi-GFP vector. Approximately 10 µg of this DNA construct was transformed to *Arabidopsis* or rice mesophyll protoplasts for transient gene expression analysis. As shown in Figure 3A and Figure 3B, strong GFP fluorescence was observed in the nucleus of arabidopsis and rice. To test the efficiency of stably expressed proteins, we inserted AtSOS2 full-length fragment into pGreen-35S-GFP/K vector by TA-based cloning to form the construct of pGreen-35S-GFP/AtSOS2-GFP, and then transformed this construct into arabidopsis by agrobacteria-mediated transformation approach. As shown in Figure 3C, we observed strong GFP fluorescence emission in the nuclei of transgenic plant root tip cells, consistent with previous reports [17], [18]. These results demonstrated the efficiency of our transient and stable vector systems.

### Expression of Proteins of Interest by the Stable Expression System

**Figure 4:**
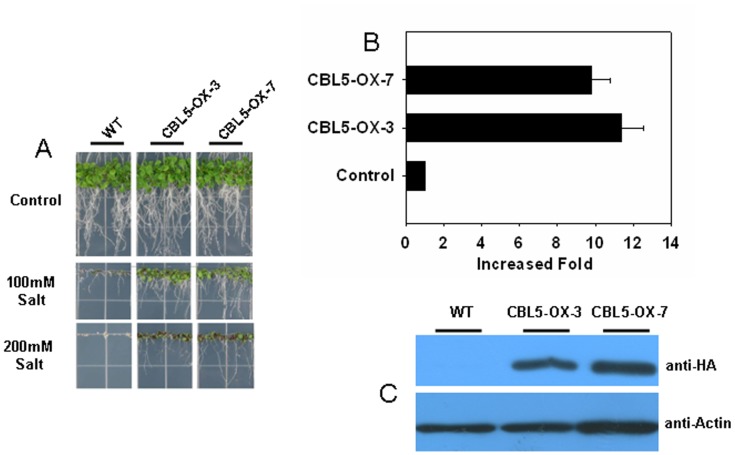
Overexpression analysis of the CBL5-HA protein by TA-based expression vectors. A) Transgenic Arabidopsis overexpressing CBL5-HA showed more tolerance to salt stress. Two individual transgenic lines overexpressing CBL5-HA (indicated by CBL5-OX-3 and CBL5-OX-7) were treated with 100 mM and 200 mM NaCl, respectively, for two weeks, after which the pictures were taken. B) Real-time quantitative PCR confirmed a higher transcriptional level of the CBL5 gene in the individual transgenic lines CBL5-OX-3 and CBL5-OX-7. C) Western blot showing the high accumulation of CBL5-HA protein in the two individual transgenic lines CBL5-OX-3 and CBL5-OX-7.

CBL5 is a calcium binding protein that can regulate the response of Arabidopsis to abiotic stress, including osmotic and drought stress [19], [20]. To validate our stable expression system, we fused the Arabidopsis CBL5 gene with the 35S promoter and an HA tag by TA cloning, allowing us to strongly express the CBL5 gene in Arabidopsis. After transformation and selection, we obtained several positive lines. Two of these lines, CBL5-OX-3 and CBL5-OX-7, showed increased tolerance to saline stress when grown in 100 mM and 200 mM saline plates (Figure 4). Similar results have been reported in previous work. Further analysis demonstrated that high levels of CBL5 transcripts accumulated in both of these lines (Figure 4B). A high accumulation of CBL5-HA was also successfully measured in these two lines (Figure 4C).

### Promoter Analysis by the Stable Expression System

**Figure 5:**
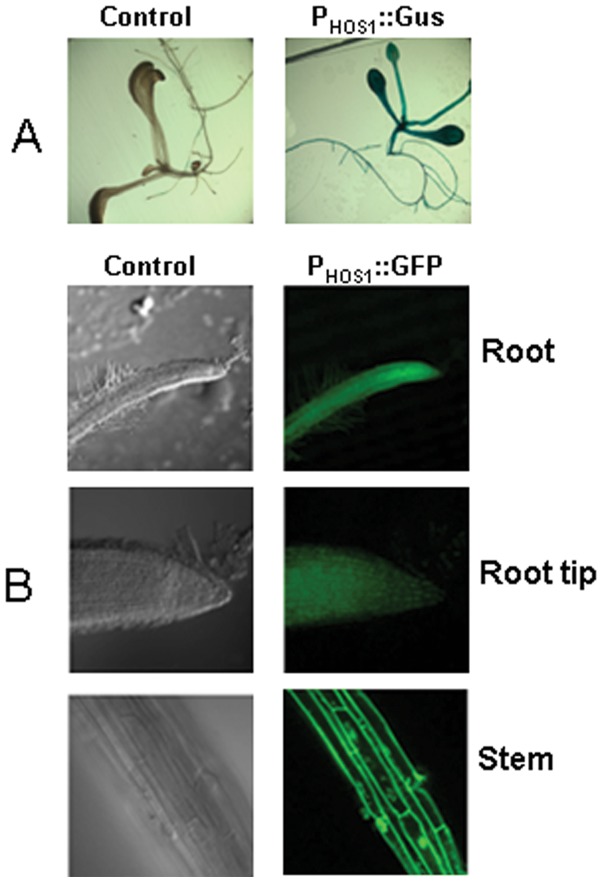
Promoter analysis using TA-based cloning vectors. A. GUS staining of an Arabidopsis line transformed with P_HOS1_::GUS, in which the HOS1 promoter was cloned into the pGreen-GUS-K vector by TA cloning. Left: The control line without transformation, Right: The transgenic line with the Tubulin promoter fused to GUS. B, GFP fluorescence of Arabidopsis transformed with P_HOS1_::3xGFP, in which the HOS1 promoter was cloned into the pGreen-3GFP-K vector by TA-based cloning. Top: The control lines without transformation. Bottom: The transgenic lines with the SOS2 promoter fused to 3xGFP.

For promoter assays, we constructed several vectors to assess the TA-based expression vectors. These vectors contained general reporter markers, such as 3xGFP and GUS. Other fluorescence proteins, including sYFP, DsRed and tdTomato, were also used to indicate the promoter intensity (Figure 2). HOS1 encodes the E3 ligase for the plant response to cold stress [21]. Here, we cloned the HOS1 protein promoter and inserted it upstream of the GUS and GFP reporter markers by the TA cloning method described above. After transformation and selection, we obtained the transgenic lines shown in Figure 4. We found that the HOS1 promoter could drive GUS (Figure 5A) or GFP (Figure 5B) expression in the root tip, lateral root primordia, vascular tissue and leaves. These results demonstrated that our vector system is efficient for promoter analysis.

### Gene Silencing Analysis by Artificial microRNA

**Figure 6:**
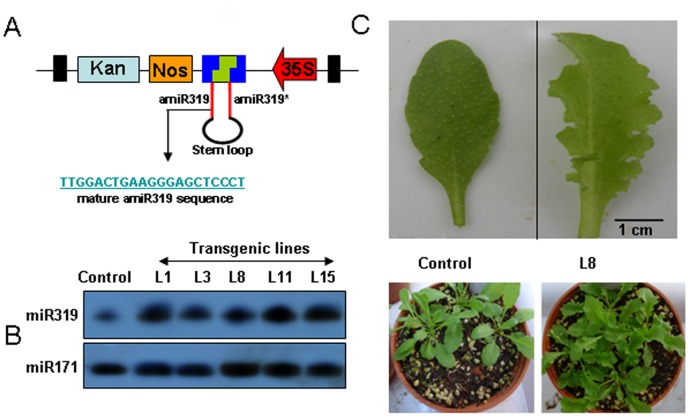
Overexpression of an artificial miR319 by the pGreen TA-based cloning system. A, Construction of an artificial miR319 by the TA-based pGreen-35S-HA-K vector. B, Detection of miR319 accumulation in five individual transgenic lines with overexpression of the artificial miR319 (indicated by L1, L3, L8, L11 and L15). C, the leaf phenotypes of the transgenic L8 line overexpressing miR319 and the control, wild type Arabidopsis. Left: The control, wild type Arabidopsis, Right: The transgenic line L8 overexpressing artificial miR319.

MicroRNAs modulate plant growth and development. Overexpression of artificial microRNAs also mimics the function of endogenous microRNAs in plant research. Schwab et al. developed an efficient approach to constructing a binary vector for overexpression of artificial microRNAs [22]. Liang et al. further improved this method [23], but these approaches still require a time-consuming procedure involving regular restriction enzyme digestion and cloning. MicroRNA319 can regulate Arabidopsis leaf shape by cleavage of its target genes from the TCP gene family, including TCP2, TCP4 and TCP10. Overexpression of endogenous Arabidopsis microRNA319 or artificial microRNA319 with normal binary vectors, such as pCAMBIA vectors, caused abnormal leaf shape [24], [25], [26]. Here, we developed our pGreen system to simplify the generation of Arabidopsis amiRNA constructs. To overexpress artificial microRNA319 in Arabidopsis in our pGreen system, we first amplified the stem loop structure of amiRNA319 by previously described methods and then inserted the amplified loop-stem loop into our pGreen system by TA cloning (Figure 6A). After Arabidopsis transformation and selection, we obtained more than 10 individual transgenic lines that accumulated high levels of miR319. Figure 6B shows that high levels of miR319 accumulated in these transgenic lines. We found that the amiRNA319 overexpression lines presented an extremely wavy leaf shape and abnormal leaf sizes (Figure 6C). Such phenotypes are consistent with previous reports, suggesting that our pGreen system is efficient in plant gene silencing and microRNA research.

### Inducible Expression Vector for Functional Gene Research

**Figure 7:**
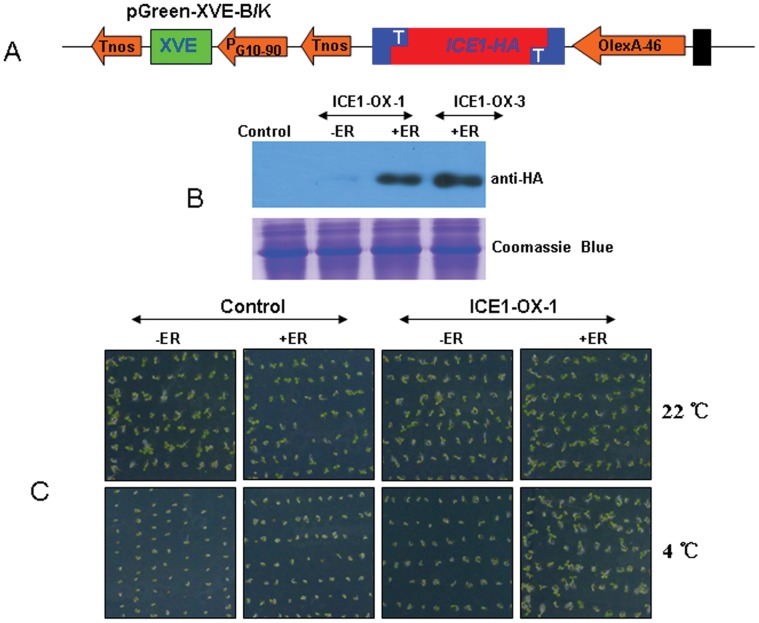
Induction of gene expression by the inducible TA-based pGreen vector system. A, Construction of the inducible expression vector with ICE1-HA by the pGreen-XVE-K vector. Only the region between the right and left borders is shown (not to scale). PG10-90, a synthetic promoter controlling XVE transcription after beta-estradiol treatment. XVE: the DNA unit encoding a chimeric fragment containing the DNA-bind domain of LexA, the transcription activation domain of VP16 and the regulatory of the human estrogen receptor; OlexA: eight copies of the LexA operator sequence; −46: the −46 35 S minimal promoter; ICE-HA; the arabidopsis ICE1 gene linking the HA tag. B, Detection of ICE1-HA fusion protein accumulation in transgenic lines (ICE1-OX-1 and ICE1-OX-3) and wild type control lines. Two-week-old Arabidopsis were either not treated or treated with 10 µM beta-estradiol for 3 days, and the ICE1-HA protein was detected by the anti-HA antibody. –ER: without beta-estradiol treatment, +ER: with beta-estradiol treatment. C, Tests of the germination capabilities of seeds from the transgenic ICE1-OX-1 line and the wild type control line at either normal 22°C conditions or at a chilling treatment of 4°C. These tests were performed for 5 days, after which photos were taken. The experiments were repeated three times with similar results, and one represent experiment is shown.

Compared with constitutive promoters, inducible promoters offer numerous advantages in functional gene analysis [26], [27]. Many inducible promoter system have already been used in plants [27], [28], [29]. Here, we also developed a set of inducible promoters based on the estrogen receptor chemical-inducible system derived from pER8 vector [27]. As shown in Figure 2, the inducer beta-estradiol strongly promoted the expression of genes of interest in pGreen-XVE-B/K vector; this vector consists of three units, the first unit contains the strong promoter of P_G10–90_ fragment, the second unit contains an activator unite named XVE, which is a chimeric transcriptional fragment consisting of the DNA-bind domain (DBD) of the bacterial repressor LexA(X; residues 1–87), the acidic transactivating domain of VP16 (V; residues 403–479), and the carboxyl region of the human estrogen receptor (E, residues 282–595). The third units contains eight copies of the LexA operator sequences fused to the -46 35S minimal promoter (OlexA-46) to control transcription of target gene. These units showed work very well in previous research [28]. To test the efficiency of this vector here, we inserted the ICE1 gene downstream of the OlexA promoter in the pGreen-XVE-K vector by TA cloning (Figure 7A). After Arabidopsis transformation and screening, we obtained several individual positive transgenic lines. As shown in Figure 7B, we found that the inducer beta-estradiol could strongly induce the protein accumulation of the ICE1 gene product in the transgenic lines ICE1-OX-1 and ICE1-OX-3, while the expression of the genes encoding such proteins could be induced in the transgenic line ICE1-OX-1 without beta-estradiol (ER) treatment. Because the ICE1 protein enhances Arabidopsis tolerance to cold stress [30], we then compared the cold tolerance capability between wild type and transgenic lines overexpressing the ICE1 protein. As shown in Figure 7C, we found that the seeds of wild type and transgenic lines exhibited good germination under normal conditions at 22°C, while the seeds of wild type and the transgenic line ICE1-HA without beta–estradiol (ER) treatment showed very weak germination capability after 5 days of chilling treatment at 4°C. However, these transgenic seeds showed good germination capability after beta–estradiol (ER) treatment, and most seeds emerged with obvious cotyledons. These results strongly supported the efficiency of our inducible vector system for functional analysis of plant genes.

## Discussion

The TA-based cloning system is very simple, highly efficient and easy to manipulate. In this study, we developed a TA-based cloning system for plant functional gene research and further validated that the TA-based expression system is highly efficient for phenotypic analysis, subcellular localization, promoter analysis, and constitutive and inducible expression. In our system, we introduced the *ccdB* gene into the TA-based expression system. Due to the lethality of the protein product of the *ccdB* gene to *E. coli* DH 5α, this approach overcomes the shortcomings of vector self-ligation, as the self-ligated vector with the *ccdB* gene cannot survive in *E. coli* DH 5α (Figure 1). Certainly, the PCR product can be inserted into the vector in either the sense or antisense orientation during TA-based cloning, but we can easily verify the insertion direction of the PCR product by special forward or reverse primers. Chen et al also used the same system to develop several vectors for plant functional gene research [10]; however, we added more vectors here. For example, we developed the vectors (pGreen-XVE-B/K) for inducible expression. We replaced the single GFP fluorescence reporter marker with 3GFP reporter marker, which is more stable and high intensity. Meanwhile, we also add other reporter markers, such as YFP, sCFP, and tDtomato, for protein subcelluar location in our system. Thus our system should further explored the application of TA-based cloning system for plant functional gene research.

The size and copy number of vectors are two limiting factors that affect ligation efficiency. It has been reported that the efficiency of *in vitro* recombination procedures is inversely proportional to the size of the vector [31]. Chen et al [10] used pCAMBIA vector as the backbone of the TA-based expression system, the size of pCAMBIA is over 12 kb, which added the difficult to molecular manipulation and ligation efficiency. Here we selected pGreen vectors that are only approximately 4.6 kb in length, as the backbone of the TA-based expression system. Moreover, the pGreen0029 vector contains the pUC replication origin, which permits high copy numbers in *E. coli*. These characteristics greatly improved the efficiency of recombination manipulation.

In conclusion, due to the higher copy and smaller size, our TA-based expression vector system provides a convenient and versatile approach for plant functional genome research.

## Supporting Information

Table S1 The prime sequences used in this article.
(DOC)
