# Supplementary material for: A Series of TA-Based and Zero-Background Vectors for Plant Functional Genomics
Source: PLoS One. 2013 Mar 29;8(3):e59576. doi: 10.1371/journal.pone.0059576 (PMC3612078; doi:10.1371/journal.pone.0059576)
Supplement: Table S1 — The prime sequences used in this article. (DOC) [file pone.0059576.s001.doc]

Table S1: the prime sequences used in this article.

| Prime Name | Temple | Prime Sequence | Notes |
| --- | --- | --- | --- |
| pUC-35S-F | pXSN | cccGAGCTCggaaacagctatgaccatgattacg | Constructing pUC-35S |
| pUC-35S-R | pXSN | cccGGTACCgaattcccgatctagtaacatagat |
| pUC-Ubi-F | pXUN | cccGAGCTCggaaacagctatgaccatgattacg | Constructing pUC-Ubi |
| pUC-Ubi-R | pXUN | cccGGTACCgaattcccgatctagtaacatagat |
| pUC-35S-GFP-F | pX-DG | cccGAGCTCggaaacagctatgaccatgattacg | Constructing pUC-35S-GFP |
| pUC-35S-GFP-R | pX-DG | cccGGTACCgaattcccgatctagtaacatagat |
| pUC-35S-DsRed-F | pX-DR | cccGAGCTCggaaacagctatgaccatgattacg | Constructing pUC-35S-DsRed |
| pUC-35S-DsRed-R | pX-DR | cccGGTACCgaattcccgatctagtaacatagat |
| pUC-35S-HA-F | pXSU-HA | cccGAGCTCggaaacagctatgaccatgattacg | Constructing pUC-35S-HA |
| pUC-35S-HA-R | pXSU-HA | cccGGTACCgaattcccgatctagtaacatagat |
| pUC-35S-Myc-F | pXSU-Myc | cccGAGCTCggaaacagctatgaccatgattacg | Constructing pUC-35S-Myc |
| pUC-35S-Myc-R | pXSU-Myc | cccGGTACCgaattcccgatctagtaacatagat |
| pUC-35S-Flag-F | pXSU-Flag | cccGAGCTCggaaacagctatgaccatgattacg | Constructing pUC-35S-Flag |
| pUC-35S-Flag-R | pXSU-Flag | cccGGTACCgaattcccgatctagtaacatagat |
| pGreen-35S-B/K-F | pCXSN | cccGAGCTCgtgctgcaaggcgattaagttgggt | Constructing pGreen-35S-B/K-F |
| pGreen-35S-B/K-R | pCXSN | cccGGTACCgaattccgatctagtaacatagat |
| pGreen-Ubi-B/K-F | pCXUN | cccGAGCTCgtgctgcaaggcgattaagttgggt | Constructing pGreen-Ubi-B/K-F |
| pGreen-Ubi-B/K-R | pCXUN | cccGGTACCgaattccgatctagtaacatagat |
| pGreen-35S-HA-B/K-F | pCXSN-HA | cccGAGCTCgtgctgcaaggcgattaagttgggt | Constructing pGreen-35S-HA-B/K |
| pGreen-35S-HA-B/K-R | pCXSN-HA | cccGGTACCgaattccgatctagtaacatagat |
| pGreen-35S-Flag-B/K-F | pCXSN-Flag | cccGAGCTCgtgctgcaaggcgattaagttgggt | Constructing pGreen-35S-Flag-B/K |
| pGreen-35S-Flag-B/K-R | pCXSN-Flag | cccGGTACCgaattccgatctagtaacatagat |
| pGreen-35S-Myc-B/K-F | pCXSN-Myc | cccGAGCTCgtgctgcaaggcgattaagttgggt | Constructing pGreen-35S-Myc-B/K |
| pGreen-35S-Myc-B/K-R | pCXSN-Myc | cccGGTACCgaattccgatctagtaacatagat |
| pGreen-35S-DsRed-B/K-F | pCXSN-DG | cccGAGCTCgtgctgcaaggcgattaagttgggt | Constructing pGreen-35S-DsRed-B/K |
| pGreen-35S-DsRed-B/K-R | pCXSN-DG | cccGGTACCgaattccgatctagtaacatagat |
| pGreen-Ubi-HA-B/K-F | pCXUN-HA | cccGAGCTCgtgctgcaaggcgattaagttgggt | Constructing pGreen-Ubi-HA-B/K |
| pGreen-Ubi-HA-B/K-R | pCXUN-HA | cccGGTACCgaattccgatctagtaacatagat |
| pGreen-Ubi-Flag-B/K-F | pCXUN-Flag | cccGAGCTCgtgctgcaaggcgattaagttgggt | Constructing pGreen-35S-Flag-B/K |
| pGreen-Ubi-Flag-B/K-R | pCXUN-Flag | cccGGTACCgaattccgatctagtaacatagat |
| pGreen-Ubi-Myc-B/K-F | pCXUN-Myc | cccGAGCTCgtgctgcaaggcgattaagttgggt | Constructing pGreen-Ubi-Myc |
| pGreen-Ubi-Myc-B/K-R | pCXUN-Myc | cccGGTACCgaattccgatctagtaacatagat |
| pGreen-Ubi-GFP-B/K-F | pCXUN-DG | cccGAGCTCgtgctgcaaggcgattaagttgggt | Constructing pGreen-Ubi-GFP |
| pGreen-Ubi-GFP-B/K-R | pCXUN-DG | cccGGTACCgaattccgatctagtaacatagat |
| pGreen-Ubi-DsRed-B/K-F | pCXUN-DR | cccGAGCTCgtgctgcaaggcgattaagttgggt | Constructing pGreen-Ubi-DsRed |
| pGreen-Ubi-DsRed-B/K-R | pCXUN-DR | cccGGTACCgaattccgatctagtaacatagat |
| pGreen-Promoter-ccdB-F | pCXSN | cccGAGCTCcgaacgatactcgaggggggatcc | Constructing pGreen-Promoter-ccdB |
| pGreen-Promoter-ccdB-R | pCXSN | cccACTAGTgaacgatcggggaaattcggatcccca |
| pGreen-3GFP-F | pPLV4 | cccACTAGTgatccatggctccaaagaagaagag | Constructing pGreen-3GFP |
| pGreen-3GFP-R | pPLV4 | cccGAGCTCgggatcggatctttacttgtacagctc |
| pGreen-GUS-F | pPLV13 | cccACTAGTccatgttacgtcctgtagaaaccc | Constructing pGreen-GUS |
| pGreen-GUS-R | pPLV13 | cccGAGCTCttgaacgatcggggatcggatcctca |
| pGreen-sYFP-F | pPLV5 | cccACTAGTccatgactagtaagggcgaggagct | Constructing pGreen-sYFP |
| pGreen-sYFP-R | pPLV5 | cccGAGCTCaatgtttgaacgatcggggatcggat |
| pGreen-sCFP-F | pPLV7 | cccACTAGTccatgactagtaagggcgaggagct | Constructing pGreen-35S-sCFP |
| pGreen- sCFP -R | pPLV7 | cccGAGCTCaatgtttgaacgatcggggatcgga |
| pGreen-tDtomato-F | pPLV10 | cccACTAGTccatgactagtaagggcgaggagct | Constructing pGreen-tDtomato |
| pGreen-tDtomato-R | pPLV10 | cccGAGCTCatgtttgaacgatcggggatcggatc |
| pGreen-OlexA-TATA-F | pLB12 | cccGAGCTCttgatcccccctcgacagcttgcatgc | Constructing pGreen-XVE-B/K |
| pGreen-OlexA-TATA-R | pLB12 | cccACTAGTgactcggtaccccctcgacacaaaaag |
| pGreen-XVE-ccdB-F | pLB12 | cccACTAGTggatccatgtacccatacgatgttcca | Constructing pGreen-XVE-B/K |
| pGreen-XVE-ccdB-R | pLB12 | cccCTCGAGgaattcccgatctagtaacatagat |
| pGreen-XVE-F | pLB12 | cccCTCGAGtgtcaaacactgatagtttaaactga | Constructing pGreen-XVE-B/K |
| pGreen-XVE-R | pLB12 | cccGGTACCttcccgccttcagtttccaagcttg |
| SOS2-GFP-F |  | atgacaaagaaaatgagaagagtgg | Construing pGreen-35S-K for overexrpession SOS2-GFP |
| SOS2-GFP-R |  | aaacgtgattgttctgagaatctctgac |
| CBL5-F |  | atgggatgtg tttgcagcaagcaattagaa | Construing pGreen-35S-K for overexrpession CBL5-HA |
| CBL5-R |  | ccggagaaaggttgggaaaatcctcgg |
| Tubulin-F |  | atgagagagatccttcacattcaagg | Construing pGreen-GUS-K for expressing tubulin promoter: GUS |
| Tubulin-R |  | agtctcataatctccctcctcttcttc |
| MiR319-F |  | tttcctcgcatctaccatcc | Construing pGreen-35S-K for overexrpession artificial miR319 |
| MiR319-R |  | gcagaaaccgaagcactttc |
| ICE1-F |  | atgggtcttgacggaaacaatggtgg | Construing pGreen-GUS-K for overexpression ICE1-HA |
| ICE1-R |  | tcagatcataccagcataccctgct |
